# Developments in Algorithms for Sequence Alignment: A Review

**DOI:** 10.3390/biom12040546

**Published:** 2022-04-06

**Authors:** Jiannan Chao, Furong Tang, Lei Xu

**Affiliations:** 1 Institute of Fundamental and Frontier Sciences, University of Electronic Science and Technology of China, Chengdu 610054, China; jn.chao@outlook.com; 2 Yangtze Delta Region Institute (Quzhou), University of Electronic Science and Technology of China, Quzhou 324003, China; furong.tang@hotmail.com; 3 School of Electronic and Communication Engineering, Shenzhen Polytechnic, Shenzhen 518055, China

**Keywords:** heuristic alignment algorithms, alignment scoring, multiple sequence alignment, alignment refinement, alignment quality estimation

## Abstract

The continuous development of sequencing technologies has enabled researchers to obtain large amounts of biological sequence data, and this has resulted in increasing demands for software that can perform sequence alignment fast and accurately. A number of algorithms and tools for sequence alignment have been designed to meet the various needs of biologists. Here, the ideas that prevail in the research of sequence alignment and some quality estimation methods for multiple sequence alignment tools are summarized.

## 1. Introduction

The developments in sequencing technologies have enabled unprecedentedly fast sequencing speeds and large-scale sequencing capabilities. The increasing number of sequences are challenging the automated sequence analysis procedures [1,2]. Sequence alignment is one of the basic tasks in the processing of biological sequences, and the accuracy of alignment affects the subsequent analyses [3]. Phylogenetics, comparative genomics, and protein structure and function prediction all depend on sequence alignment to look for conserved regions [4,5,6,7].

Sequence alignment software usually inserts gaps between the nucleotides or amino acid residues in the sequences, so that as many similar sites as possible can be aligned. Finally, a character matrix with the same number of columns and rows that correspond to the number of the sequences is obtained. In this review, the pairwise sequence alignment algorithms and the corresponding scoring system, heuristic algorithms for multiple sequence alignment and their defects, and quality estimation methods used to test multiple sequence alignment software are reviewed. There have been several reviews for multiple sequence alignment, such as Refs [8,9]. In order to be distinct from the previous work, this review will try to present a general overview of the algorithms that prevail in this field and cover the work of the last several years.

## 2. Pairwise Sequence Alignment

Pairwise sequence alignment is the basis of multiple sequence alignment and mainly divided into local alignment and global alignment. The former is to find and align the similar local region, and the latter is end-to-end alignment. A commonly used global alignment algorithm is the Needleman–Wunsch algorithm [10], which has become the basic algorithm that is used in many types of multiple sequence alignment software. The algorithm usually consists of two steps: one is calculating the states of the dynamic programming matrix; and the other is tracking back from the final state to the initial state of the dynamic programming matrix to obtain the solution of alignment. Time and space complexity of pairwise sequence alignment algorithms based on dynamic programming is *O*(*l*_1_*l*_2_), where *l*_1_ and *l*_2_ are the lengths of the two sequences to be aligned. Such overheads are acceptable for short sequences but not for sequences with more than several thousand sites. As a space-saving strategy of the dynamic programming algorithm, the Hirschberg algorithm [11] is able to complete alignment by the space complexity of *O*(*l*) without any sacrifice of quality.

An optimal solution for the pairwise sequence alignment of very long sequences is usually impossible to find in practice. Heuristic algorithms can be used to reduce the time and space cost incurred by dynamic programming. For this, the most widely applied method is to limit the state transition and conduct the alignment in a smaller search space. Although heuristic algorithms do not guarantee that there will be no poor results, ideal alignments can be achieved in many types of software because the sequences to be aligned are usually quite similar.

In addition, the hidden Markov model (HMM) is also widely utilized in sequence alignment tools, such as HHalign [12], which can perform high accurate profile HMM alignment. In terms of sequence alignment, an HMM is a statistical model that describes probability distribution over biological sequences. According to the three problems that are interesting when using HMMs [13], the adoption of HMMs in sequence alignment has three corresponding issues: the scoring problem, the alignment problem, and the training problem [14]. The first two problems are about how likely a given HMM could generate a sequence and how the HMM could produce the corresponding alignment, and the third problem is about how to build the structure and estimate the parameters of the HMM based on given sequences, which could be either aligned or unaligned. The scoring parameters are implicitly trained, and thus, the alignment methods based on HMMs do not rely on explicit scoring systems, which makes them independent of the empirical scoring methods described in Section 2.3. HMM-based sequence alignment methods are gaining popularity nowadays. COVID-Align [15], one of the efforts to combat the COVID-19 pandemic, is an online alignment tool based on a profile HMM [16] estimated using HMMER [17] and about 2500 high-quality SARS-CoV-2 genomes. Additionally, MAGUS + eHMMs [18], an alignment tool for fragmentary sequences, chooses eHMM rather than MAFFT-addfragments [19], which adopts the Smith–Waterman algorithm [20], to add sequences to the backbone alignment because of its better performance in terms of alignment accuracy.

### 2.1. Divide and Conquer

**Figure 1:**
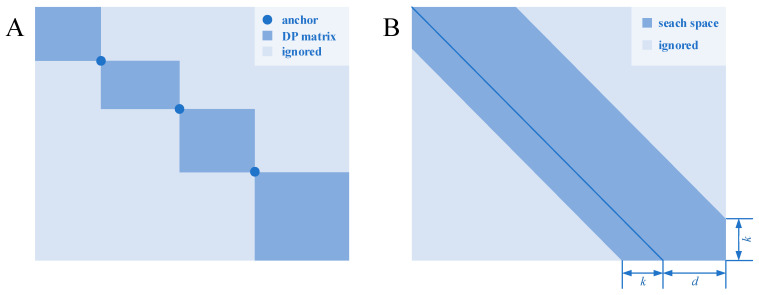
Two heuristic algorithms for pairwise sequence alignment. (**A**) A dynamic programming matrix, which is separated by several anchors, which is certain to be in the optimal path. (**B**) A shape-based bounded dynamic programming matrix in which the light blue block is calculation-free because these states are thought to be less likely to be in the optimal path.

One of the heuristic methods is based on divide and conquer. In such methods, homologous segments (or seeds) are found and used as the “anchors” for the alignment. Each anchor point can divide the dynamic programming matrix into four sub-matrices located at the four corners. Backtracking always goes toward the upper left direction, and these anchors are regarded as the waypoints that the optimal path must pass; therefore, the sub-matrices located at the lower left and upper right are useless and naturally disregarded. When more anchor points distributed throughout the sequences are found, the scale of the dynamic programming matrix can be greatly reduced, thereby reducing the time and space complexity (Figure 1A).

Many alignment algorithms are designed to find such seeds to accelerate alignment. FASTA [21,22] and BLAST [23] are two of the classic ones. They both search for common substrings in a way similar to the Rabin–Karp algorithm [24]. Moreover, some other software, such as MUMmer [25,26], uses data structures, such as suffix tree [27], suffix array [28], or FM-index [29], to find homologous segments. Recently, Minimap2 [30], which also adopts this strategy, is gaining popularity and is used as the basis of ViralMSA [31], which can perform multiple sequence alignment of viral genomes with the help of a reference sequence and is linearly scalable with the number of sequences. MAFFT [32] utilizes fast Fourier transform (FFT) to accelerate the calculation of the correlation between two sequences, which is an indicator of homologous regions of the sequences. These homologous regions can then act as anchors in pairwise sequence alignment. Additionally, it is concluded in Ref [32] that the accuracy of FFT-based methods is almost unaffected by this heuristic approach.

The concept of divide and conquer is also adopted in some multiple sequence alignment software to scale to large datasets, such as FAME [33] and FMAlign [34], which vertically divide the sequences using common seeds among the sequences, and MAGUS [35] and Super5 [36], which divide the sequences horizontally into subsets that are small enough to be aligned fast and accurately.

### 2.2. Bounded Dynamic Programming

Bounded dynamic programming [37] is based on a heuristic idea: if two sequences have close similarity, then the number of gaps inserted in the sequences during alignment will be relatively small. Therefore, the possible backtracking paths will be close to the diagonal of the dynamic programming matrix for similar sequences. The possible interval can be seen as a strip with certain width parallel to the diagonal (Figure 1B). The states located in the strip are calculated, while the others are ignored. The width of the strip reflects the trade-off between the alignment accuracy and time consumption: a wide strip means more states needed to be calculated, whereas a narrow strip means that more states could be ignored, which will, however, increase the possibility of missing the optimal path.

Several methods are available for determining the strip range. A basic idea is to use the shape-based division, but this does not fully consider the biological significance and is rarely used. A simple improvement of this method is to set a threshold to filter the states that could be ignored. If the score of one state in the dynamic programming plus the score for the transition from this state to the final state is greater than the threshold, then transitions from this state are allowed. However, this approach requires transition scores to be estimated and a threshold to be set. The transition scores can be conservatively estimated by its upper bound, and the threshold can be generally determined using the iterative method proposed in Ref [38].

### 2.3. Scoring System of Pairwise Sequence Alignment

The most critical factor for the quality of a pairwise sequence alignment is the scoring system. It is the basis of the sequence alignment, including multiple sequence alignment, because it determines the direction of the alignment and reflects its quality. Most types of the sequence alignment software aim to obtain good alignment by defining an explicit or implicit objective function for scoring and improving their ability to achieve high score by adjusting alignment strategy. The higher score an alignment can achieve, the higher we think its accuracy will be in the corresponding scoring system. As an example, a model and the corresponding scoring system for pairwise alignment of nucleotide sequences containing frameshifts and stop codons comprise the main feature of MACSE, a multiple sequence alignment tool that is specific to coding sequences and takes into account frameshifts and stop codons [39].

Generally, the score of a pairwise sequence alignment is the sum of the scores of all aligned pairs. For alignment of two protein sequences, for example, each pair of aligned sites is scored depending on whether a gap is involved, or, if no gaps are involved, whether the two aligned residues are matched or mismatched. When a gap is involved, a gap penalty, which is usually a negative score, is given. Additionally, the score for matched and mismatched amino acid residues is generally determined using a substitution matrix.

The most basic substitution matrix is based on whether the two residues are matched or not. More complex matrices also take into consideration the attributes of nucleotides or amino acid residues. For example, the score of conversion–transversion matrix reflects the difference between conversion and transversion frequency in natural mutations [40]. Protein sequences contain more types of residues than nucleic acid sequences, and therefore, the substitutions and the frequency involved are more complicated. Early amino acid substitution matrix is based on the properties and the codons of amino acids [41]. More recent amino acid substitution matrices rely on the analysis of the substitution frequency of a large number of homologous sequences [41] and aim to reflect the natural probability of substitutions among amino acids at certain evolutionary distances by giving conservative substitutions higher scores. Typical amino acid substitution matrices are percent accepted mutations (PAM) [41,42] and BLOcks SUbstitution matrix (BLOSUM) [43]. Although these matrices are widely adopted, there are also matrices designed for specific protein domain, such as GPCRtm, which is summarized from the transmembrane segments of the G-protein-coupled receptor (GPCR) rhodopsin family for the reason that it is not optimal to align sequences with marked compositional biases using the general-purpose matrices [44].

In addition to substitution matrices, gap penalty is also an important part of the scoring system. A simple rule is to assign a fixed negative score when a nucleotide or amino acid residue aligns with a gap. However, this scoring method has some intrinsic limitations, mainly because insertions and deletions (indels) are small-probability events, especially in nucleic acid sequences where indels can cause frameshifts and disrupt all subsequent codons. Once a gap is inserted in an alignment, adjacent gaps are more likely to occur compared with gaps inserted at a distance from the first gap. Therefore, almost all sequence alignment algorithms now use the gap penalty rule based on the number of the gaps successively inserted, and the most typical one is the affine penalty. No optimal solution is universally applicable for the gap penalty, which is referred to as a “black art” requiring constant trial of errors [45].

## 3. Multiple Sequence Alignment

Optimal alignment results can be obtained under a certain scoring system with the multi-dimensional dynamic programming by extending the classic Needleman–Wunsch algorithm. However, the time and space requirements grow exponentially with the number of sequences, which makes it impossible to complete an alignment in an acceptable time or space using this method even for a small sequence data set. To overcome this problem, some heuristic algorithm frameworks that seek quasi-optimal alignment solutions within reasonable time and space have been developed. There are also important algorithms that cannot be categorized as one of the algorithm frameworks described below, such as MAHDS [46], which is partially based on tandem repeat search algorithms [47,48] and is able to produce statistically significant alignments from highly divergent nucleotide sequences, while the other methods could hardly cope with these kinds of data sets.

### 3.1. Star Alignment Strategy

**Figure 2:**
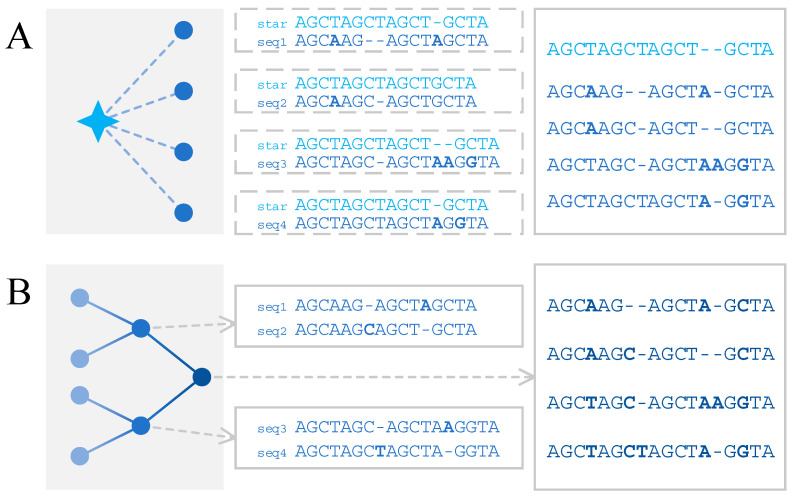
Two strategies for multiple sequence alignment. (**A**) Star alignment strategy performs multiple sequence alignment based on the consistencies among pairwise alignments of the sequences pending alignment and a center sequence, which is in light blue. (**B**) Progressive alignment strategy performs multiple sequence alignment along a pre-built guide tree, each of whose internal nodes represents an alignment of two sequences, one sequence with one profile, or two profiles.

The star alignment strategy [49] is a heuristic method that aligns multiple sequences by the consistencies among pairwise alignments of each of the sequences to be aligned and a center sequence, which could be either generated or selected from the input (Figure 2A). It is based on the assumption that given sequence ***a***, ***b***, ***c***, and pairwise alignments *A*(***a***, ***c***), *A*(***b***, ***c***), if there is ***a****_i_* ~ ***c****_k_* in *A*(***a***, ***c***) and ***b****_j_* ~ ***c****_k_* in *A*(***b***, ***c***), then ***a****_i_* should be aligned with ***b****_j_*, where ***c*** is regarded as the center sequence, and ***a****_i_* ~ ***b****_j_* means that the *i*th residue of ***a*** is aligned with the *j*th residue of ***b***.

The star alignment algorithm uses the star topology to simulate the relationship between sequences, and the selection of the center sequence determines the accuracy of the alignment to some extent. A brute-force choice is the sequence that has the shortest average distance from the other ones, but the time required is proportional to the square of the number of the sequences. Therefore, it is usually difficult to apply this scheme to data sets with large number of sequences. Generally, the longer the sequence is, the more likely the information lost by other sequences due to mutations will be retained. On the basis of this assumption, the clustering software CD-HIT [50] selects the longest sequence in each possible cluster as the cluster center. HAlign [51], which adopts star alignment strategy to perform multiple sequence alignment, also selects the longest sequence as the center.

This system has a limited ability to simulate the relationship between sequences, so it is less effective for data sets with complex relationships; but good alignment results can often be obtained for data sets with high similarities. Moreover, thanks to its extremely low time complexity, it can handle tasks that are almost impossible to be completed by other algorithms within a finite time, so that an approximate solution can be quickly obtained. The pairwise sequence alignments involved in the star alignment strategy are independent of each other, making it easy to parallelize programs based on the star alignment strategy, for example MASC [52] and I-CSA-M [53], which is able to utilize multiple mainframes simultaneously.

### 3.2. Progressive Alignment Strategy

The progressive alignment strategy [54] is another heuristic method, which performs multiple sequence alignment based on a pre-built guide tree (Figure 2B). Each leaf node in the guide tree represents a sequence, and each internal node represents an alignment of the sequences corresponding to its descendant leaf nodes (also known as profile). Two sequences (two leaf nodes), a sequence and a profile (a leaf node and an internal node), or two profiles (two internal nodes), are sequentially aligned along the paths from the leaf to the root. When the alignment proceeds with the root node, the multiple sequence alignment result is obtained.

The tree topology is used in the progressive alignment algorithm to simulate the relationships between sequences, and therefore, the alignment quality depends on the quality of the guide tree. Several established hierarchical clustering algorithms for the construction of guide trees are available. Among them, unweighted pair group method with arithmetic mean (UPGMA) [55] has been the most widely used. However, because UPGMA depends on the distance matrix of the sequences, the *O*(*n*^2^) time and space complexity is still a problem, where *n* represents the number of the sequences to be aligned. To avoid the calculation of the distance matrix, some multiple sequence alignment software adopts heuristic methods, such as PartTree [56] used in MAFFT [32] and mBed [57] used in ClustalO [58] and Kalign 3 [59]. On the other hand, there are kinds of string distances for the estimation of similarities between two sequences. Several of them, such as edit distance and Damerau–Levenshtein distance, have a time complexity of *O*(*l*^2^), which is too time consuming. Therefore, the string similarity based on *k*-mers [42] has become popular because of its lower time complexity. In addition, it is worth noting that simple chained guide trees, even in a random order, might help to produce more accurate alignment of protein families of a large number of sequences for structure-based benchmarks [60] and reduce the time consumption of the guide tree construction. The effect of chained guide trees has been discussed in Refs [61,62,63].

In addition, PRANK [64] and PAGAN [65] implemented a phylogeny-aware progressive alignment algorithm, which takes into consideration the difference between the insertions and deletions and makes them especially suitable for producing alignments for evolutionary analyses, at a cost of poor performance on structural benchmarks. ProPIP [66,67] goes further by the adoption of an explicit mathematical model describing the evolution of indels.

Compared with the star alignment, the tree topology used in the progressive alignment fits far more closely with the natural relationships among sequences; therefore, progressive alignment can often process more complex data [68] and has become the core of algorithms used in most multiple sequence alignment software. Both star alignment and progressive alignment try to transform a multiple sequence alignment problem to approximately *n* pairwise alignments. However, the pairwise alignments in the latter involve profile-to-profile alignment, which requires additional computation. Furthermore, the selection of the center sequence in star alignment could be very simple, while the construction of a guide tree in progressive alignment requires a hierarchical clustering, which is complicated and thus takes much more time. These make progressive alignment more time consuming than star alignment, especially when the number of sequences is over tens of thousands.

## 4. Defects of Heuristic Algorithms and Countermeasures

The star alignment and the progressive alignment strategies have two main defects. The “once a gap, always a gap” cardinal rule [69] is one of the problems; once a wrong gap is inserted in the alignment, it will continue to exist and affect all the subsequent alignment. Although this rule causes the gap error to spread, it does not create a baseless error, and the root of the error lies in another defect, i.e., the local optimum trap. Local optima usually arise from the greedy principle. Specifically, the optimum selected for a particular sub-problem is not necessarily optimal for the overall problem, and this will adversely affect the final alignment. For these two heuristic methods, every pairwise sequence alignment process, regardless of whether a profile is involved, aims to find a local optimal solution, and therefore, the multiple sequence alignment, which is pieced together based on these local optima, will inevitably have the possibility of falling into a local optimum trap. The star alignment is more affected by this defect because of its limited system ability compared with progressive alignment.

Two methods can be applied to handle these problems [70]: one is consistency objective function, which aims to reduce the probability of introducing errors in the alignment stage, and the other is iterative refinement, which aims to remove any errors in the final alignment by post-processing.

### 4.1. Consistency Objective Function

Consistency objective function provides another scheme for scoring two aligned residues. For example, consider three sequences, ***a***, ***b***, and ***c***, for which the pairwise sequence alignments are *A*(***a***, ***b***), *A*(***a***, ***c***), and *A*(***b***, ***c***). If there is ***a****_i_* ~ ***b****_j_* in *A*(***a***, ***b***) and ***b****_j_* ~ ***c****_k_* in *A*(***b***, ***c***), then the score of ***a****_i_* ~ ***c****_k_* should be increased in the multiple sequence alignment of these three sequences. Match scores of every possible pair are re-estimated in this way. Gap penalties are already considered in the pairwise sequence alignment. The matching score of the two residues obtained by re-estimation with the consistency objective function is the score after the gaps have been penalized. Therefore, gap penalties need not be considered in the subsequent multiple sequence alignment process. It takes *O*(*n*^2^*l*^2^) time to obtain all the possible pairwise alignments and *O*(*n*^3^*l*) time to re-estimate the match scores of all the pairs indirectly aligned [71], which is impractical for large data sets. The consistency objective function was proposed by Notredame et al. and has already been implemented in multiple sequence alignment software T-Coffee [71,72]. ProbCons [73] goes further by the adoption of HMM, a more formal probabilistic framework. The concept of consistency is also utilized to combine several alignments into a better alignment, which is implemented in M-Coffee [74].

### 4.2. Iterative Refinement

The iterative refinement method [54,75] processes the results of multiple sequence alignment to remove errors caused by the local minimum trap and the “once a gap, always a gap” rule. There are several ways to perform the iterative refinement, two of which will be introduced in this section.

Progressive alignment relies on the guide tree, but the heuristic distance estimation or hierarchical clustering do not necessarily produce the optimal tree for alignment. Therefore, in some iterative refinement algorithms, the completed alignment results are used to recalculate the sequence distance matrix to construct a more solid guide tree, which can be used to improve the alignment performance in an additional round of alignments. This refinement method is seldom used as the core of iterative refinement because of its excessive time overhead. Nevertheless, SATé [76,77] uses this iterative technique to meet the challenge of highly accurate alignment estimation on data sets with hundreds of sequences.

Currently, an iterative refinement method based on division and re-alignment is widely used. In this method, the results of the old completed alignment are divided horizontally into two parts, and the columns that contain only gaps are deleted to form a valid alignment for each part. Then, the two parts are re-aligned to get new completed alignment. Finally, the old and new completed alignments are compared, and the better one is selected as the basis for the next iteration. The iteration will continue until it meets a certain condition, which is usually provided as an argument for users to determine, depending on how much time they can afford to improve the accuracy of the alignment. A commonly used horizontal segmentation method is to cut an edge of the guide tree to divide all sequences into two parts, which has been shown to produce the best trade-off results between time consumption and accuracy [78,79]. Moreover, FAMSA divides an alignment based on whether a gap is present in a certain column and is claimed to be able to improve the alignment quality for data sets up to 1000 sequences [80]. There are also methods that vertically divide a completed alignment, such as SpliVert [81].

A reliable scoring method is needed to quickly and effectively compare the two completed alignments. The SP (sum of pairs) [82,83] score is commonly used. SP sums the scores of all-against-all pairwise alignments of all the sequences (the scores of the pairwise sequence alignments are calculated as described in Section 2.3). Generally, the alignment of two gaps is ignored, so the score will be 0. A brute-force way to calculate the SP score of an alignment is costly, but some efficient algorithms are available, such as Ref [84], which is based on the pre-computation of the gap interval information of each input sequence.

Another important kind of multiple sequence alignment methodology is stochastic and mainly based on evolutionary algorithms, which are intrinsically iterative. It starts with a set of possible alignments and performs genetic operators on them to yield a new generation of alignments, which act as the initial alignments of the next iteration. This kind of algorithm has become a promising alternative field of multiple sequence alignment research [8]. Two of the methods that adopt evolutionary algorithms are MO-SAStrE [85] and its parallel version M2Align [86], which aim to optimize multiple objective functions simultaneously (while the classic methodologies optimize only one objective function). Recent research has shown the ability of genetic algorithms to combine and improve the quality of several quasi-optimal alignments [87] (while traditional methods, such as SA-GA [88], tend to start from random alignments).

## 5. Quality Estimation of Multiple Sequence Alignment Software

Multiple sequence alignment software uses different algorithms, and therefore, the alignment quality can vary. To score the alignment algorithms and to provide references for the design and development of sequence alignment software, different quality estimation methods have been proposed. Several kinds of classical quality estimation methods [89], as well as their pros and cons based on the desirable properties proposed in Ref [90], are discussed in this section and summarized in Table 1.

### 5.1. Estimation Based on Reference Alignment

Structured benchmarks for protein alignment software include BAliBASE [91], which is one of the most commonly used benchmarks. These benchmarks provide fixed test sets and reference alignments that have been manually or automatically refined based on the three-dimensional structure of proteins, with the assumption that amino acid residues corresponding to the same position in the three-dimensional structure should be aligned. Although BAliBASE and other similar benchmarks that provide fixed test sets and reference alignments are widely used, if they are not regularly updated, the developers of multiple sequence alignment software may tend to optimize their software only on limited data sets, resulting in the “high in score but low in ability” phenomenon.

Another method scores multiple sequence alignment software using generated data sets. These methods simulate the evolution of sequences (i.e., substitution or indel of residues) using a probabilistic model and generate the evolved sequences and the reference alignment based on the evolution. Because the generated mutations are completely determined by the evolution model, the accuracy of such benchmarks is restricted by the degree to which the adopted model represents the natural evolution. In some conditions, the probabilistic model can even bias the estimation if it is similar to the sequence relationship model the tested software adopts, which also poses a challenge to the establishment and selection of the probabilistic model.

These two types of benchmarks provide preset reference alignment against which the performance of newly developed software can be tested. Therefore, a scoring method is needed to measure the degree to which the alignment of the tested software is close to the reference. Two commonly used scoring methods are the sum of pairs (SP) and total column or true column (TC) scores [92], which estimate the similarity of two alignments by counting the number of common pairs and common columns in the two alignments. The Friedman rank test [93,94] and the Wilcoxon signed-rank test [32,80] could be used for alignment accuracy discrimination by reporting a *p*-value, which indicates the likelihood that the performance difference between different methods is due to chance.

**Table 1:** Quality estimation methods of multiple sequence alignment software.

	Structural Benchmark	Simulated Sequences	Commonality-Based
Scalability	Low	High	High
Pre-Built Alignment	Yes	Yes	No
Scoring Methods	Sum of pair score and true column score	Sum of pair score and true column score	Multiple overlap score and head-or-tail score
Dependency	Protein structure	Probabilistic model	/
Test Sets	Fixed	Configurable	Not limited
Drawbacks	Limited to the diversity of benchmarks	Adopted model may have defects	Tested software can make common mistakes
Examples	BAliBASE [91] Pfam [95] HOMSTRAD [96]	ROSE [97] INDELible [98] Dawg [99]	MUMSA [100]

### 5.2. Estimation Based on the Commonality among Alignments by Different Software

Another kind of quality estimation method does not rely on reference alignment but the commonality among the alignments obtained by different multiple sequence alignment strategy. It is based on the idea that if several different pieces of software consistently align two residues, then, most likely, these two sites are correctly aligned. However, an important defect of this method is that if different pieces of software consistently but wrongly align two residues, then this error will be deemed correct in the scoring. Unlike the estimation methods based on reference alignment, the commonality-based methods use some special scoring methods, such as the multiple overlap score [100] and the head-or-tail (HoT) score [101]. The multiple overlap score considers that every aligned pair in an alignment could get a higher score if the pair appears in more alignments. The HoT score assumes that a good sequence alignment tool should not have the assumption about the direction of sequences, which means that it should produce two consistent alignments based on sequences in the original and the reversed order.

## 6. Conclusions

The explosive growth of biological sequence data has brought unprecedented challenges to the automated biological sequence analysis software. Sequence alignment, as the basis of sequence analysis, restricts subsequent analysis in applicability and accuracy. In this review, pairwise sequence alignment and its scoring system, main algorithms for multiple sequence alignment, as well as their advantages and disadvantages, and the quality estimation methods for multiple sequence alignment software, are presented and discussed. The collation of this information (Supplementary Table S1) might help the researchers of sequence alignment and the users of sequence alignment tools.

## Supplementary Materials

The following supporting information can be downloaded at: https://www.mdpi.com/article/10.3390/biom12040546/s1, Table S1: The overview of tools and methods mentioned in the review.
