# Supplementary material for: Developments in Algorithms for Sequence Alignment: A Review
Source: Biomolecules. 2022 Apr 6;12(4):546. doi: 10.3390/biom12040546 (PMC9024764; doi:10.3390/biom12040546)
Supplement: Supplementary file 1 [file biomolecules-12-00546-s001.zip › biomolecules-1614149-supplementary.pdf]

Supplementary Table S1. The overview of tools and methods mentioned in the review.

Tools or Methods	Typical Features	Working on	Year
**HHalign**	pairwise alignment of profile HMMs	protein sequences	2005
**COVID-Align**	pre-built HMM	COVID sequences	2021
**MAGUS+eHMMs**	MAGUS and HMM	fragmentary sequences	2021
**MAFFT-addfragments**	designed for adding fragmentary sequences to an alignment	fragmentary sequences addition	2012
**MUMmer**	suffix trees and maximal unique matching subsequences	pairwise alignment of DNA sequences of very large scale	1999
**Minimap**	seed-chain-align	pairwise alignment for nucleotide sequences	2016
**ViralMSA**	massively scalable	viral genomes	2021
**MAFFT**	FFT	general use	2002
**FAME**	vertical division	general use	2020
**FMAlign**	vertical division	nucleotide sequences	2021
**MAGUS**	horizontal division and Graph Clustering Merger	large-scale alignments	2020
**Super5**	horizontal division	large datasets	2021
**mBed**	embedding methods	rough guide tree construction	2010
**PartTree**	length-based clustering	rough guide tree construction	2006
**MACSE**	frameshifts and stop codons	coding sequences	2011
**GPCRtm**	specific to GPCR	scoring systems for GPCR	2015
**MAHDS**	sophisticated mathematical process	highly divergent nucleotide sequences	2021
**HAlign**	suffix trees	sequences of very large scale	2015
**MASC**	suffix trees and Spark Parallel Framework	nucleotide sequences	2017
**chained guide trees**	simple topology	protein families of large number of sequences for structure-based benchmarks	/
**PRANK**	phylogeny-aware progressive alignment	alignments for evolutionary analyses	2008
**PAGAN**	phylogeny-aware progressive alignment	alignments for evolutionary analyses	2012
**ProPIP**	Poisson Indel Process (explicit mathematical model)	alignments for evolutionary analyses	2021
**T-Coffee**	consistency objective function	general use	2000
**ProbCons**	Probabilistic consistency	protein sequences	2005
**M-Coffee**	consistency objective function	alignments combination	2006
**SATé**	iteration between re-aligning and tree re-construction	simultaneous alignment and tree estimation for DNA	2009
**FAMSA**	division strategy based on gaps for iterative refinement	thousands of protein sequences	2016
**SpliVert**	vertical division strategy for iterative refinment	refinement of protein sequence alignments	2020
**MO-SAStrE**	multi-objective metaheuristic	general use	2013
**M2Align**	multi-objective metaheuristic and its parallelization	general use	2017
**SAGA**	genetic algorithms	general use	1996
